# Expediting the transition from replacement medicine to tissue engineering

**DOI:** 10.1093/rb/rbw014

**Published:** 2016-03-17

**Authors:** Arthur J. Coury

**Affiliations:** Department of Chemical Engineering, Northeastern University, Boston, MA, USA

**Keywords:** tissue engineering, medical devices, combination products, regenerative medicine

## Abstract

In this article, an expansive interpretation of “Tissue Engineering” is proposed which is in congruence with classical and recent published definitions. I further simplify the definition of tissue engineering as: “Exerting systematic control of the body’s cells, matrices and fluids.” As a consequence, many medical therapies not commonly considered tissue engineering are placed in this category because of their effect on the body’s responses. While the progress of tissue engineering strategies is inexorable and generally positive, it has been subject to setbacks as have many important medical therapies. Medical practice is currently undergoing a transition on several fronts (academics, start-up companies, going concerns) from the era of “replacement medicine” where body parts and functions are replaced by mechanical, electrical or chemical therapies to the era of tissue engineering where health is restored by regeneration generation or limitation of the body’s tissues and functions by exploiting our expanding knowledge of the body’s biological processes to produce natural, healthy outcomes.

## Introduction

With increasing knowledge of the body’s physiological processes, attention of the medical therapy community is shifting from replacing body parts and processes to controlling biology in ways that promote desired physiology and anatomy. This transition is encompassed in the concept of tissue engineering. Several definitions of the term have been published since it came into prominence. For example, Langer and Vacanti, pioneers in the field, published the following definition: ‘Tissue engineering is an interdisciplinary field that applies the principles of engineering and life sciences toward the development of biological substitutes that restore, maintain, or improve tissue function’ [1]. Williams current definition is: ‘Tissue engineering is the creation of new tissue for the therapeutic reconstruction of the human body, by the deliberate and controlled stimulation of selected target cells through a systematic combination of molecular and mechanical signals’ [2]. In its broad manifestation, I define tissue engineering as: ‘Exerting systematic control of the body’s cells, matrices and fluids.’ [3]. By this definition, it conforms to the spirit of the other published definition statements.

## Scope of tissue engineering

From an industrial perspective, it is worthwhile to consider products already in use, those in development and those in concept. The world market in medical products (pharmaceuticals, devices, combination products, biologics) through 2014 was ∼$2 trillion [4]. Actual and projected breakdown of this ‘Life Sciences’ sector of the world economy by one analysis comprises: Pharmaceuticals ($1.23 trillion, 2014; $1.61 trillion, 2018); Biotech ($289 billion, 2014; $445 billion, 2019); Medical Technology ($364 billion, 2013; $514 billion, 2020) [4]. Pharmaceuticals and biotech products comprise about 80% of the global Life Sciences sector. Within this broad sector, there is some blending of categories, such as drug/device and biologic/device combination products, estimated to produce revenues of $151 billion by 2020 [5]. While tissue engineering, in the broad sense defined above, can be effected within any of the Life Sciences categories, a very conservative analysis of its scope eliminates synthetic pharmaceuticals ($1.23 trillion in 2014) and biopharmaceuticals ($160 billion in 2014) [6] from this tissue engineering consideration. Of the remaining ∼25% of the medical products market (∼$500 billion), by the broad interpretation of tissue engineering, 70–80% of such medical products are still replacement products (artificial joints, heart valves, pacemakers, intraocular lens, dental restorations, transplanted allogeneic tissues, etc.) ($350–$400 billion) and 20–30% are tissue engineering products (∼$100–150 billion). The latter figure, while controversial in the scale of its interpretation, signifies that tissue engineering is currently a very successful component of the medical products industry. Such products range from wound treatments to guided tissue regeneration products *in situ* (e.g., surgical adhesion prevention, oral tissue healing) to stem cells to organ and tissue production *in vitro* (e.g., cartilage, dermal regeneration). This trend toward supplanting replacement medical products with tissue engineering products is inexorable and desirable although inconsistent and subject to inaccurate predictions of timing and scale.

## Examples of tissue engineering products

To support my position that many medical products, not normally considered as tissue engineering products are, in fact such, several examples are provided. My former company, Focal, Inc. developed and commercialized the first surgical sealants to achieve regulatory approval, specifically, a lung sealant approved in the U.S. and Europe, and a dural sealant approved in Europe. These poly(ethylene oxide) (PEG)-based tissue adherent resorbable compositions involved a dual-redox/photopolymerization application process [7]. In preclinical animal studies, a dural excision rabbit model was studied. The PEG hydrogel was used to seal a 4 mm^2^ excision of the rabbit dura mater above the brain. Examination of the treatment zone 10 days later revealed a confluent, vascularized multi-layer neo-membrane seal [3] covering the whole gap. This treatment with result is a form of tissue engineering called ‘guided tissue regeneration.’ In our experience, this healing phenomenon of generation of tissue on a synthetic or natural substrate by cell migration and/or mitosis under a resorbing hydrogel surface has been observed in dermal wound healing, surgical adhesion prevention and lung sealing as well as dural sealing. In these examples of what I call tissue engineering, no extrinsic cell production, ingrowth-promoting scaffold or bioactive pharmacologic factor was used.

Two other examples of control of tissue response, i.e., of tissue engineering, without extrinsic cell/scaffold/factor employment are: tissue expansion techniques [8] and topical products for dermal scar revision [9,10]. The former technique involves implantation of a saline-expandable balloon under the skin and above the muscle layer in targeted zones and periodically expanding the balloon to induce the skin to expand in area. The consequent expanded tissue can be used to produce cosmetically favorable appearance in areas in need of reconstruction such as the breast, scalp, hands, arms and legs [8]. The dermal scar reduction therapies employ either silicone gels [9] or hydrogels [10] applied to surgical or traumatic scars to reduce their mass and appearance over a period of time. While small molecule drugs are sometimes incorporated into the gels, the result appears mainly be due to extended hydration and a mechano-transduction effect.

Tissue engineering products that fall into a more narrow interpretation of the published definitions (i.e., employing extrinsically-produced cells, ingrowth scaffolds, with or without bioactive molecules) include several examples of existing products or those reaching ‘proof of concept’ that represent a growing transition from ‘replacement medicine’ to ‘tissue engineering.’ An established engineered tissue product, developed by Genzyme Corporation, is the ‘Autologous Chondrocyte Implant, (ACI)’ [11]. This product, launched in 1995 is the first FDA-approved product of its kind and involves treating a zone of injured knee hyaline cartilage with cultured cells expanded from a biopsy of healthy cartilage removed from a non-articulating section of the cartilage and covering the transplant with a periosteum membrane. An advance in this cell-based cartilage regeneration approach, marketed by Genzyme Corporation, is the development of a product that incorporates a scaffold to deliver the autologous cultured chondrocytes called ‘MACI^®^’ (Matrix-Induced Autologous Chondrocyte Implant) [12]. Advantages of this technique include elimination of periosteum excision and transplantation and potential amenability to laparoscopic delivery. Approved for sale in Europe and Australia, it is being developed for worldwide use by the company, Vericel, after divestiture by Genzyme [13].

Porcine small intestinal submucosa (SIS) is a decellularized membrane pioneered by Badylak that has been used widely in clinical practice in applications such as hernia repair, shoulder repair and other body locations [14]. It resorbs in the body within several months with concomitant generation of local ingrowth and replacement of the implant volume with tissue. The treated SIS contains intrinsic active growth factors to promote tissue formation. The degradation and tissue generation occur with significant inflammation [15], which generally resolves with favorable clinical outcome. The technique involves implantation of scaffold alone without added cells, and represents tissue engineering in the broad sense.

Advanced approaches to *in vitro* generation of organs or organ segments employ all of the tools of tissue engineering: cells, scaffolds and bioactive molecules. The laboratory of Anthony Atala is a leader in translating this technology to the clinic. Early work on generation of ureter segments and complete bladders *in vitro* with autologous cells provided successful clinical therapies validated in multi-year studies [16] and led to the formation of the tissue engineering company, Tengion, which carries on the development of organ regeneration therapies, with lead therapies being ‘neo-urinary conduit’ and ‘neo- kidney augment’ [17].

Lior Gepstein of the Technion, Israel, has reported on the production of cardiomyocytes from human embryonic stem cells and manifestation of rate-responsive cardiac rhythm control in rat and pig models [18]. This tissue engineering technology, when further developed, shows promise for supplanting the utilization of the electronic pacemaker. These transformed cells are injected without using a scaffold.

A long-sought goal of tissue engineering has been the regeneration of autologous hair follicles. Significant recent progress has been made using both embryonic and adult stem cells modified in culture and transplanted subcutaneously in the murine model. The follicles survive, integrate with surrounding tissue, undergo hair cycles and exhibit piloerection and other normal follicular processes. Human hair follicles were successfully transplanted to the nude mouse as well [19].

The trajectory of tissue engineering is following a typical scenario exemplified by ultimately successful product initiatives such as antibodies, biopharmaceuticals, certain replacement devices, surgical techniques, even prescription drugs, where initial promising results lead to over-exuberance and the hard realities such as resourcing, technical setbacks and public opinion lead to setbacks and retrenchment. The field of tissue engineering is currently on the upswing where large device and drug companies are committing resources to the field, often as defensive measures and small companies dedicated to focused products in the field are forming with realistic goals derived from earlier experiences of failed companies. For example, Medtronic, Inc. is maintaining its lead in spine therapy products with the help of its ‘Infuse’ bone morphogenic protein product (delivered on collagen sponge) used to enhance the rate of spinal fusion in conjunction with its metal lumbar fusion cage [20]. Genzyme/Sanofi Aventis, a company basic in enzyme therapy for rare diseases, was the leader leader in cell therapy for cartilage repair and large-area skin regeneration [21], but recently divested its cell therapy products to the company: Aastrom Biosciences, recently renamed Vericel [13]. Vericel is actively developing and marketing a range of living cell-based products and has assumed a leadership role in tissue engineering.

## Summary

It is appropriate, in this summary to consider the importance of biomaterials in the gradual transition of therapies from replacement medicine to therapeutic tissue engineering. Of course, replacement medical devices were composed largely of structural materials used in contact with body tissues. They were usually delivered to the body using tools of entry such as scalpels, curettes, saws, adhesives etc. Biomaterials such as sutures and staples were used for closure often with the aid of needles, dispensers, etc. For tissue engineering, scaffolds, gels and other devices are used to deliver cells and bioactive factors, all of which may comprise the therapy. Even if biomaterials are not part of the therapy imparted, adjunctive devices comprised of biomaterials will still be essential to deliver the therapy. Therefore the types and functions of biomaterials may change, but they will always be crucial to all stages of advancement of medical therapy. Continuing development of biomaterials is necessary to promote advances in both replacement and tissue engineering therapy.

In conclusion, the trajectory of tissue engineering in the broad sense advances generally upward and significant products are emerging. Such progress is resulting in the supplanting of replacement therapeutic products with engineered tissue or strategies to control the body’s functions to induce favorable therapeutic outcomes. This transition is generally positive in that it will result in the regeneration, generation, limitation or healing of tissue toward its healthy, potentially durable state. While replacement products indicated above are highly biomaterials-based, tissue engineering products are also dependent on biomaterials as components of their composition or for adjunctive devices to deliver the therapies. As new business opportunities and as defensive measures, emerging companies dedicated to the field and those founded on pharmacologic and device premises are currently devoting significant resources to developing tissue engineering products.

*Conflict of interest statement*. None declared.
